# Systematic Identification of Housekeeping Genes Possibly Used as References in *Caenorhabditis elegans* by Large-Scale Data Integration

**DOI:** 10.3390/cells9030786

**Published:** 2020-03-24

**Authors:** Jingxin Tao, Youjin Hao, Xudong Li, Huachun Yin, Xiner Nie, Jie Zhang, Boying Xu, Qiao Chen, Bo Li

**Affiliations:** 1 College of Life Sciences, Chongqing Normal University, Chongqing 401331, China; taojingxin@cqnu.edu.cn (J.T.); haoyoujin@hotmail.com (Y.H.); lixudong.cqnu@gmail.com (X.L.); huachun-yin@cqnu.edu.cn (H.Y.); xinernie.cqnu@gmail.com (X.N.); zhangjiejuej@126.com (J.Z.); xbywh@126.com (B.X.); 2 Scientific Research Office, Chongqing Normal University, Chongqing 401331, China; chenqiao.cqnu@gmail.com

**Keywords:** microarray, housekeeping gene, reference gene, *Caenorhabditis elegans*, *RankAggreg*

## Abstract

For accurate gene expression quantification, normalization of gene expression data against reliable reference genes is required. It is known that the expression levels of commonly used reference genes vary considerably under different experimental conditions, and therefore, their use for data normalization is limited. In this study, an unbiased identification of reference genes in *Caenorhabditis elegans* was performed based on 145 microarray datasets (2296 gene array samples) covering different developmental stages, different tissues, drug treatments, lifestyle, and various stresses. As a result, thirteen housekeeping genes (*rps-23*, *rps-26*, *rps-27*, *rps-16*, *rps-2*, *rps-4*, *rps-17*, *rpl-24.1*, *rpl-27*, *rpl-33*, *rpl-36*, *rpl-35*, and *rpl-15*) with enhanced stability were comprehensively identified by using six popular normalization algorithms and *RankAggreg* method. Functional enrichment analysis revealed that these genes were significantly overrepresented in GO terms or KEGG pathways related to ribosomes. Validation analysis using recently published datasets revealed that the expressions of newly identified candidate reference genes were more stable than the commonly used reference genes. Based on the results, we recommended using *rpl-33* and *rps-26* as the optimal reference genes for microarray and *rps-2* and *rps-4* for RNA-sequencing data validation. More importantly, the most stable *rps-23* should be a promising reference gene for both data types. This study, for the first time, successfully displays a large-scale microarray data driven genome-wide identification of stable reference genes for normalizing gene expression data and provides a potential guideline on the selection of universal internal reference genes in *C. elegans*, for quantitative gene expression analysis.

## 1. Introduction

Genome-wide expression analysis has always played a crucial role in the field of the functional genome. However, the data generated by high-throughput RNA-sequencing (RNA-seq) and microarray requires an authentic tool for validation [1]. Quantitative real-time PCR (qPCR) has been widely used for validating gene expression data due to its high sensitivity, rapid execution and specificity [2,3]. However, the reliability of qPCR reactions is inevitably affected by the quality and integrity of RNAs, the efficiency of cDNA synthesis, and PCR efficiency [4,5,6]. To accurately quantify gene expressions in different spatial-temporal conditions, reference genes are widely used as internal controls to minimize the misinterpretation of expression data.

The free-living nematode *Caenorhabditis elegans* has been widely used as a model organism for a range of studies, spanning from development to drug discovery. Gene expression analysis in this model organism constitutes a powerful tool to discover new roles for different types of molecules. Traditionally, a set of housekeeping genes encoding actin (*act-1*) [7,8], tubulin (*tba-1*) [9,10,11], glyceraldehyde-3-phosphate dehydrogenase (*gpd-2*) [12,13], translation initiation factor 3C (*eif-3.C*) [14,15], calsequestrin (*csq-1*) [13,16], Rho GTPase (*cdc-42*) [17,18,19,20], and peroxisomal membrane protein related (*pmp-3*) [18,21] were thought to be appropriate reference genes for the normalization of gene expression in *C. elegans*. However, some reports indicated that the transcription levels of these conserved reference genes may be changed under different conditions, such as developmental stages, drug treatments, and hypoxia [22,23]. Therefore, selecting such biased reference genes can lead to misinterpreting qPCR results, and consequently, output misleading expression data.

With the development of sequencing and microarray technology, analyzing RNA-seq and microarray data allows to identify reliable reference genes in diverse organisms such as *Homo sapiens* [24], *Setaria viridis* [25], *Malus* × *domestica* Borkh [26], *Mizuhopecten yessoensis* [27], *Gossypium hirsutum* [28], *Ciona savignyi* [29], *Streptomyces coelicolor* [30], *Arabidopsis thaliana* [31], and so on. However, the main focus of these studies was to evaluate a set of known reference gene candidates for the stability of expression using several bioinformatics algorithms: geNorm [32], RefFinder (http://www.leonxie.com/referencegene.php), NormFinder [33], BestKeeper [34], and delta-Ct method [35]. Additionally, in all the above studies, the identification of suitable reference genes was based on tightly controlled experimental conditions, whereas it is supported that the expression of these genes can vary in different tissues under different experimentally controlled conditions. Indeed, strictly speaking, an ideal reference gene should be expressed universally among different tissues and stably under different conditions.

Despite the availability of extensive microarray and RNA-seq data in *C. elegans*, an unbiased genome-wide search from the large-scale datasets to identify reliable reference genes is still lacking. For this reason, to obtain accurate results for systematic gene expression analysis, 145 publicly available microarray datasets encompassing a wide range of biological and experimental conditions, including different developmental stages, different tissues, drug treatments, lifestyles, and various stresses (microwave and toxic chemicals exposure, pathogen infection, hypoxia, starvation, and so on), were systematically analyzed to identify stable reference genes. Subsequently, the stability of 13 newly identified and 13 commonly used reference genes was further validated using six independent gene expression datasets. The validated genes here are expected to serve as potential resources for further investigating molecular mechanisms under diverse experimental conditions using the qPCR method.

## 2. Materials and Methods

### 2.1. Data Collection and Filtration

Theoretically, an ideal housekeeping gene (HKG) or reference gene should be expressed constantly in all cell types and under various experimental conditions. To obtain universal, robust and reliable HKG candidates possibly used as reference genes in *C. elegans*, datasets obtained under various experimental conditions were retrieved from Gene Expression Omnibus (GEO) (http://www.ncbi.nlm.nih.gov/geo/). Considering the consistency of experimental results, only the published microarray datasets from GPL200 [Celegans] Affymetrix *C. elegans* Genome Array were used for further analysis. Annotation information of microarray used in matching probes with corresponding genes was also downloaded from GEO database. To describe genes in detail, more annotation data from Wormbase database (https://wormbase.org/), Gene Ontology Consortium (http://geneontology.org/), and Interpro (https://www.ebi.ac.uk/interpro/) were mapped to probe sets in Genome Array.

All downloaded microarray datasets were filtrated according to the following criteria: (1) the total number of samples is more than three in an individual microarray dataset; (2) no duplicated samples in other datasets; and (3) the raw data in *.CEL format is available in GEO.

### 2.2. Methodology

#### 2.2.1. Normalization of Microarray Data

**Figure 1:**
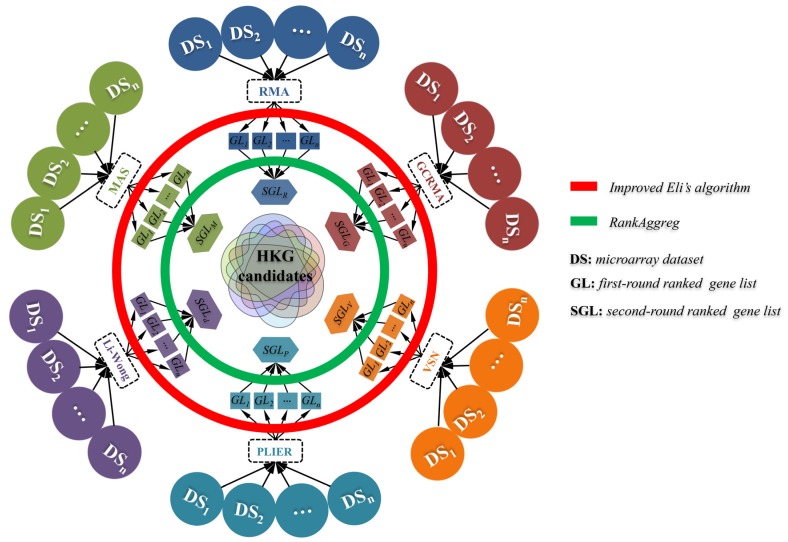
The workflow for the identification of housekeeping gene (HKG) candidates. Briefly, gene expression matrix (GEM) for each microarray dataset was obtained by normalizing raw data using six algorithms (*RMA*, *MAS5*, *Li-Wong*, *GCRMA*, *PLIER*, and *VSN*, respectively), and the corresponding first-round ranked gene lists (abbr. GL, from GL_1_ to GL_n_) were achieved based on gene expression stability. And then, multiple GLs derived from the algorithm-specific normalization were merged as the second-round ranked gene list (abbr. SGL) using *RankAggreg* algorithm and produced six SGLs (SGL_R_, SGL_M_, SGL_d_, SGL_G_, SGL_P_, and SGL_V_). Finally, these six SGLs were intersected to obtain the final reliable HKG candidates.

Figure 1 shows the methodology used in this study. Raw signal intensities for each probe set in the CEL files were analyzed using a series of algorithms implemented in R and Bioconductor package, including *Robust Multiarray Analysis* (*RMA*) [36], *Affymetrix Microarray Suite version 5* (*MAS5*) [37], *Cheng Li and Wing Hung Wong’s method* (*Li–Wong*) [38], *GeneChip RMA* (*GCRMA*) [39], *Probe Logarithmic Intensity Error* (*PLIER*) [40], and *Variance Stabilization Normalization* (*VSN*) [41].

(1) *Robust Multiarray Analysis (RMA)*. *RMA* is the most widely used preprocessing algorithm for Affymetrix and Nimblegen gene expression microarray [42]. For *RMA* normalization, the raw intensity values of microarray are background corrected, log_2_-transformed, and then quantile-normalized in order. Then a linear model is fitted to the normalized data to obtain an expression measure for each probe set in each array.

(2) *Affymetrix Microarray Suite version 5* (*MAS5*). *MAS5* is a sensitive algorithm to normalize and summarize the standard probe level [37], which offers a robust and non-logarithmic mean value generated by subtracting the MisMatch (MM) value from their Perfect-Match (PM) values. Thus, the increased variation is easily observed at low signal strength.

(3) *Cheng Li and Wing Hung Wong’s method* (*Li–Wong*). *Li–Wong* is a commonly used algorithm to compute gene expression levels of microarray data [38], which normalizes microarrays using an invariant set of genes and then fits a parametric model to the probe set data, without background correction in data preprocessing.

(4) *GeneChip RMA* (*GCRMA*). As an improved *RMA* algorithm, *GCRMA* is popular for preprocessing microarray data [39]. This normalization strategy is achieved by calculating a background adjustment that ignores the MisMatch (MM) intensities but incorporates sequence information from the probes (GC content).

(5) *Probe Logarithmic Intensity Error* (*PLIER*). This method can improve signal value by accounting for experimentally observed patterns in probe behavior and handling error at the appropriately low and high signal values. It is assumed that the error is proportional to the PM intensity without background correction and log-transformation.

(6) *Variance Stabilization Normalization* (*VSN*). *VSN* is a non-linear method that aims to keep the variance constant over the entire data range. It comprises data calibration, the quantification of differential expression, and the quantification of measurement error. The *VSN* normalized data is not logged again for comparisons based on logarithmic intensities of the data.

#### 2.2.2. First-Round Ranking of Gene Expression Stability

After the data normalization using the specific algorithm, top 5000 robustly expressed genes with the lowest standard deviations were chosen according to the following three criteria derived from the improved Eli’s approach: (1) gene expressions must be detected in all tissues and under various conditions; (2) genes were sorted based on the increased variance across various tissues and experimental conditions; and (3) no exceptional expression in any single tissue or experimental conditions for each gene. It means that log_2_-expression value is not significantly different from the averaged log_2_-normalized expression level by two-fold or more.

#### 2.2.3. Second-Round Ranking of Gene Expression Stability

To obtain more stable reference genes, the first-round ranked gene lists (GLs) derived from normalized data using the specific algorithm (such as *RMA*) were integrated into a corresponding second-round ranked gene list (such as SGL_R_) by *RankAggreg*, which is the most popular algorithm to perform aggregation of ordered lists based on the ranks via the Cross-Entropy Monte Carlo algorithm or the Genetic Algorithm [43]. Similarly, five SGLs (SGL_M_, SGL_L_, SGL_G_, SGL_P_, and SGL_V_) were obtained.

#### 2.2.4. Identification of HKG Candidates Used as Reference Genes

After data normalizing and gene ranking, six second-round ranked gene lists, including SGL_R_, SGL_M_, SGL_L_, SGL_G_, SGL_P_, and SGL_V_, were intersected to identify the most reliable HKG candidates. A gene is considered as a reliable HKG candidate when it appears in at least three SGLs.

### 2.3. Functional Annotation and Enrichment Analysis

Functional annotation is crucial for understanding the biological functions and mechanisms, and therefore Gene Ontology (GO) was used for this purpose in this study. It arranges annotation terms in a hierarchical manner and thus makes annotations in a gene list amenable to automated analysis. Moreover, the gene enrichment analysis was performed to explore the biological processes and signal pathways in which housekeeping genes may participate, for the union of the six groups (top 50 of SGL_R_, SGL_M_, SGL_L_, SGL_G_, SGL_P_, and SGL_V_). All gene function annotations in this study were performed by Bioconductor package org.Ce.eg.db and biomaRt [44,45], enriched and visualized by Metascape [46] and Cytoscape (v3.7) [47], respectively.

### 2.4. Evaluation of HKG Candidates

#### 2.4.1. Evaluation of HKG Candidates Using Newly Published Datasets

The *Gini coefficient* (*GC*) has been used to quantify the inequality of gene expression levels across different and experimental conditions [48] and is very helpful for identifying housekeeping genes [49]. To investigate the stability of gene expression, two independent indexes, *Gini coefficient* (*GC*) and *standard deviation* (*SD*) were adopted in this study. The *GC* is calculated as follows:

(1) $$GC=\frac{2\sum_{i=1}^{n}ix_{i}}{n\sum_{i=1}^{n}x_{i}}-\frac{n+1}{n}$$

where

*GC*: Gini coefficient;

$x_{i}$: the gene expression value in the i-th sample;

*n*: sample numbers.

The *SD* is analyzed as follows:

(2) $$\sigma =\sqrt{\frac{1}{n}\overset{n}{\underset{i=1}{\sum }}{\left(x_{i}-\bar{x}\right)}^{2}}$$

where

σ: standard deviation of gene expression values;

$x_{i}$: the gene expression value in the i-th sample;

*n*: sample numbers.

#### 2.4.2. Comparison of Newly Identified HKG Candidates and Commonly Used Reference Genes

To compare the reliability of HKG candidates identified in this study with commonly used reference genes (CRGs), the expression levels of 13 genes (*cyc-1*, *tba-1*, *atp-3*, *mdh-1*, *gpd-2*, *eif-3.C*, *act-1*, *cdc-42*, *pmp-3*, *act-2*, *csq-1*, *ama-1*, and *rbd-1*) were validated using the independent microarray and RNA-seq datasets. The microarray datasets from Affymetrix Inc. and Agilent Inc. were normalized using *RMA* and *log-quantile*, respectively. For RNA-seq datasets, the log_2_(CPM+1) was used to normalize read counts for gene expression level comparison [50].

## 3. Results

### 3.1. Large-Scale Microarray Datasets Collection

By the end of 2018, 171 GEO series from microarray GPL200 platform had been released in GEO database. Among them, 26 datasets were excluded because of sample duplication, no raw data or a smaller sample size (*n* < 3). Finally, 145 datasets containing 2296 high-quality samples were used for further analysis. The detailed information is listed in Supplementary Material Figures S1–S3.

### 3.2. Data Normalization and Gene Probe Matching

All 145 microarray datasets were normalized to gene expression matrices using the *rma* (for *RMA*), *mas5* (for *MAS5*), *expresso* (for *Li–Wong*), *gcrma* (for *GCRMA*), just*Plier* (for *PLIER*), and *vsnrma* (for *VSN*) from R packages as *affy*, *gcrma*, *plier*, and *vsn*, respectively. After data normalization, each gene expression matrix (GEM) contained 22,652 probes (rows in GEM) which correspond to 23,556 genes (rows in GEM). After the filtration of missing values, a total of 21,112 rows were kept in GEM. When one probe was matched to multiple genes, it was deleted. If a gene was matched by more than one probe, its expression level was measured by the median of gene expression values. Finally, 16,724 genes and their expression values were obtained.

### 3.3. First-Round Ranking Based on Gene Expression Stability

For each GEM, 16,724 genes were ranked based on parameter *SD*. Considering the feasibility of datasets and the computing power, the top 5000 genes in each ranked gene list (GL) were used for the subsequent analysis. Detailed information of all GLs containing top 5000 genes obtained by six normalization algorithms is shown in Supplementary Material Figure S4.

### 3.4. Second-Round Ranking Using RankAggreg

To get a more reliable gene list, all 145 first-round ranked gene lists were aggregated into a single ranked gene list using the *RankAggreg* algorithm. Top 50 genes from this single ranked gene list were selected to generate the second-round ranked gene list (SGL). All SGLs are shown in Supplementary Material Figure S5.

### 3.5. Overlapping for Determining the HKG Candidates Used as Reference Genes

**Figure 2:**
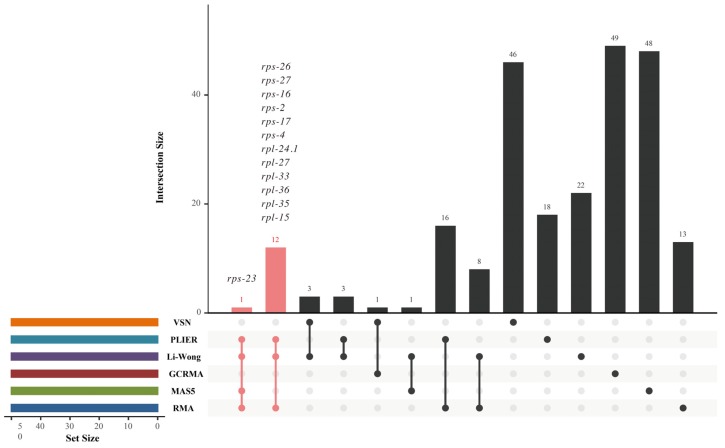
Intersection analysis of top 50 genes from each SGL.

Due to the inconsistency of gene ranking analysis, a reliable HKG candidate is considered when it is included in at least three SGLs. In total, 13 HKG candidates were identified and are listed in Table 1, of which *rps-23* was shared by SGL_R_, SGL_M_, SGL_L_, and SGL_P_, while *rps-27*, *rps-16*, *rps-26*, *rps-4*, *rps-2*, *rps-17*, *rpl-24.1*, *rpl-15*, *rpl-35*, *rpl-36*, *rpl-27*, and *rpl-33* were shared by SGL_R_, SGL_L_, and SGL_P_ (Figure 2). These results also show that 32 genes were shared by two SGLs. However, the majority of genes are specific to one SGL.

### 3.6. Gene Annotation and Enrichment

After merging, 241 genes were obtained from six SGLs (50 genes/each). Among them, 13 HKG candidates were identified as the most reliable reference genes, including seven genes encoding small ribosomal proteins (*rps-23*, *rps-27*, *rps-16*, *rps-26*, *rps-4*, *rps-2*, and *rps-17*) and six genes encoding large ribosomal proteins (*rpl-24.1*, *rpl-15*, *rpl-35*, *rpl-36*, *rpl-27*, and *rpl-33*).

**Figure 3:**
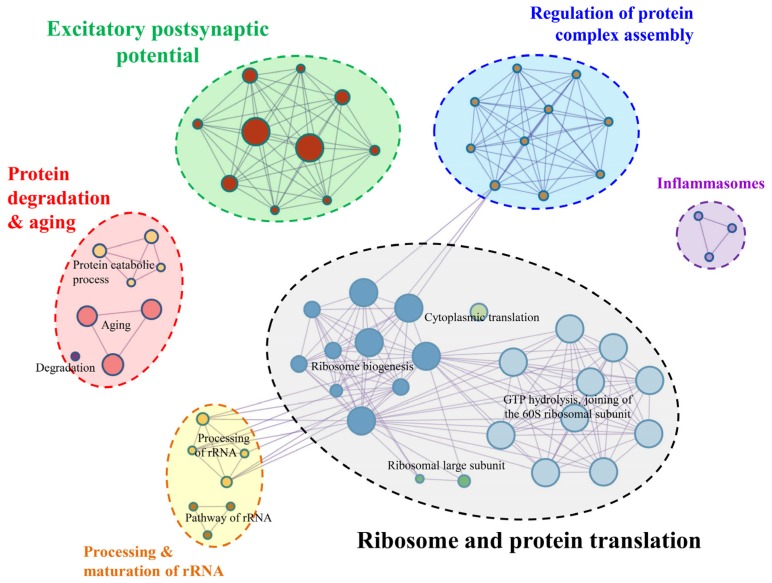
Function enrichment of all top 50 genes in six SGLs.

Annotation analysis shows that the majority of genes are associated with ribosome, protein synthesis, protein degradation, protein processing and transporting, and neuronal synapses and signal transmission. Enrichment analysis of KEGG pathway, GO biological process, and Reactome Gene Sets generated 12 clusters with an enrichment factor ≥ 1.5, gene count ≥ 3 and the *p*-value < 0.01. Membership similarity analysis reveals that these genes are mainly involved in GTP hydrolysis and assembly of the 60S ribosomal subunit, ribosome biogenesis and aging (Supplementary Material Figure S6). To better show their functional relations, statistically significant terms were selected to represent the corresponding clusters (Figure 3).

By a manual literature survey, these enriched terms were grouped into six main categories: ribosome and protein translation, excitatory postsynaptic potential, protein degradation and aging, regulation of protein complex assembly, processing and maturation of rRNA, and inflammasomes (Figure 3).

### 3.7. Validation of HKG Candidates Used as Reference Genes

**Figure 4:**
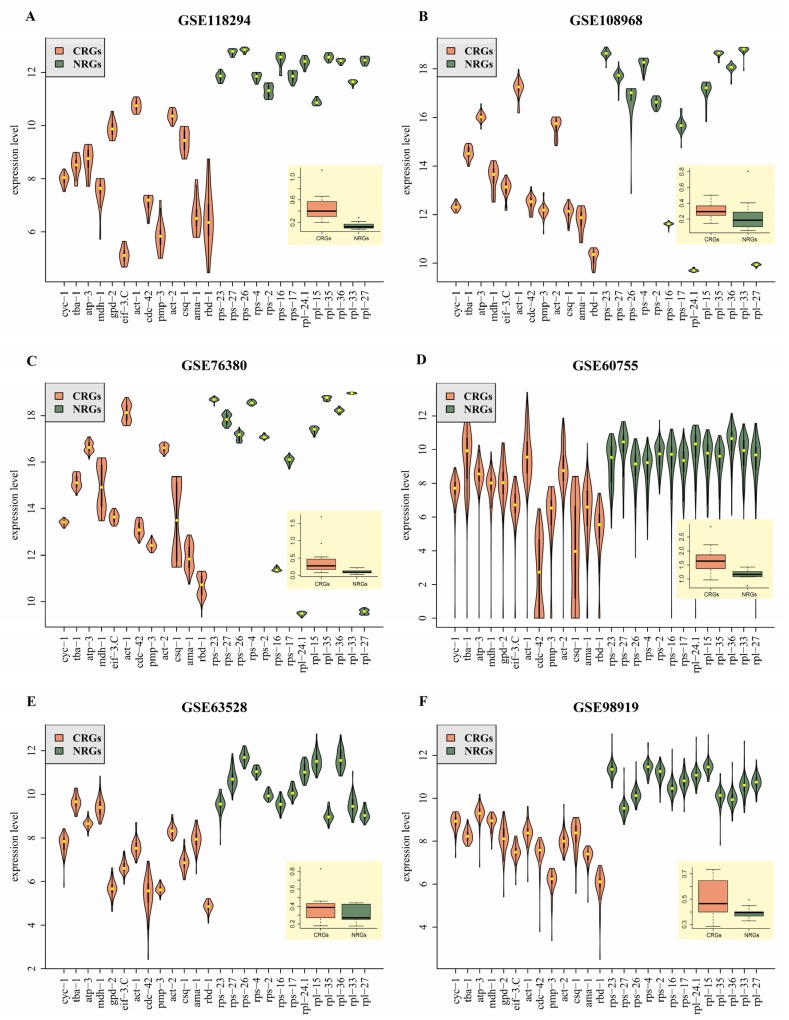
Violin plots showing the reliability of expression levels and boxplots representing standard variations in six independent datasets, for 13 NRGs (dark green) and 13 CRGs (orange). (**A**–**C**) and (**D**–**F**) represent validated results by using three microarray datasets and three RNA-seq datasets, respectively.

To confirm the reliability of 13 HKG candidates identified in this study, the validation was performed using three newly published microarray datasets (GSE118294 [51], GSE108968 [52], and GSE76380 [53]) and three RNA-seq datasets (GSE63528 [54], GSE60755 [55], and GSE98919 [56]) (Table 2). The validation result of dataset GSE118294 revealed that the variation of standard deviation (*SD*) of 13 newly identified reference genes (NRGs) (dark green boxplot in Figure 4A) was much lower than that of commonly used reference genes (CRGs) (orange boxplot in Figure 4A). Similar results were also obtained for dataset GSE108968 (Figure 4B), GSE76380 (Figure 4C), GSE60755 (Figure 4D), and GSE98919 (Figure 4F). It is worth noting that *SD* variations of CRGs and NRGs were not statistically different, but the former was slightly more unstable than the latter for dataset GSE63528 (Figure 4E). In summary, gene expression levels of the 13 HKG candidates potentially used as reference genes are stable.

### 3.8. Comparison between HKG Candidates Identified in This Study and Commonly Used Reference Gene

To further confirm the availability of 13 HKG candidates used as reference genes, gene expression profiles of 13 NRGs and another 13 CRGs were compared (Table 2 and Figure 4). To better show the comparison results, the top 10 genes among all 26 genes (13 NRGs + 13 CRGs) were selected for further analysis. As shown in Table 2, the frequency of occurrence of NRGs in the top 10 genes is higher (≥ 60%) than that of CRGs based on *SD* value and *GC* ranking. Additionally, their expression levels are more stable than those of CRGs (Figure 4). For one given test dataset (such as GSE118294 in Figure 4A), the variations of expression levels for 13 NRGs (dark green violins and boxplot) are generally lower than another 13 CRGs. Similar results were also found in the other five test datasets (Figure 4B–F), although the pattern is not very obvious in the GSE63528 (Figure 4E).

## 4. Discussion

Despite the availability of extensive transcriptome data in *C. elegans*, to our best knowledge, the systematic analysis of large-scale gene expression datasets for the identification of stable reference genes has not been conducted. This work aims to identify housekeeping gene candidates potentially used as reference genes for quantitative gene expression analysis. Theoretically, HKGs are constitutively expressed in all tissues and experimental conditions to maintain the basal cellular functions. They are characterized by wide distribution and constant and high expression. In this study, 13 HKG candidates potentially used as reference genes for gene expression analysis in *C. elegans* were identified by large-scale data integration and systematic analysis.

### 4.1. Data Integration and Its Advantages

Data integration has been widely used in omics studies, which can facilitate information mining from large-scale omics data [57]. For transcriptome data, there are two methods widely used for data integration: direct data merging and result combining after the analysis. In this study, the latter was applied to screen HKG candidates used as reliable reference genes. For the direct data merging method, batch effects cannot be avoided. To remove batch effects, the real biological differences may also be removed, which can compromise the integration and interpretation of the data [58,59].

The integration of multiple transcriptome datasets has been advocated to increase the sample size and maximize the statistics power, such as reducing the probability of false-negative results [60]. Therefore, 145 microarray datasets obtained from diverse developmental stages, tissues or experimental conditions were selected to identify reliable reference genes from HKG candidates for quantitative gene expression analysis in *C. elegans*.

### 4.2. Why Microarray Datasets Were Adopted?

With the development of high throughput expression technologies (microarray and RNA-seq), massive gene expression datasets are produced daily. There is a good agreement between RNA-seq and microarray relative to gene expression, despite some data variability in low-expression genes that may be due to the difference in expression platform and data analysis [61,62]. Microarray data were used in this study by considering the following reasons: (1) microarray is a mature genomic platform with a well-established data analysis pipeline; (2) microarray is reliable in model organisms [63] and we just focus on well-annotated genes in model organism *C. elegans*; and (3) despite the fact that RNA-seq enables to identify non-coding differentially expressed genes or not well-annotated genes that offer a potential for improved mechanistic clarity, we do not consider those genes. Therefore, DNA microarray datasets in GEO database were used for the identification of reliable reference genes for gene expression analysis in *C. elegans*. Additionally, to reduce the heterogeneity, only the datasets from GPL200 (Celegans) Affymetrix *C. elegans* Genome Array were selected, which is the most popular microarray platform used by thousands of studies of *C. elegans* worldwide [64].

### 4.3. Importance of Normalization Methods

For omics data analysis, the normalization of raw data is one of the most pivotal steps [65], which significantly affects the reproducibility of data and the reliability of experimental results [66]. Similarly, for microarray data, the normalization is helpful for removing biases and increasing the accuracy of the analysis [67]. Available normalization methods for microarray data generally assume a similar global expression pattern among samples being studied. Currently, there are many algorithms used for microarray data normalization, such as *RMA*, *MAS5*, *Li–Wong*, *GCRMA*, and so on. However, data normalized by diverse methods shows distinct shapes of statistical distributions and even leads to totally different analysis conclusions. To obtain more credible and pervasive conclusions, six widely used algorithms (*RMA*, *MAS5*, *Li–Wong*, *GCRMA*, *PLIER*, and *VSN*) for microarray data were applied in this study.

### 4.4. Reliability of Newly Identified HKG Candidates Possibly Used as Reference Genes

An ideal reference gene should be stably, highly, and universally expressed in tissues or cells regardless of the histology, pathological conditions, or cellular physiological-metabolic states [57,68]. Therefore, the large-scale transcriptome datasets under various kinds of conditions were integrated to identify HKG candidates, which will be potentially used as reliable reference genes for quantitative gene expression studies in *C. elegans*. The systematic analysis revealed that 13 HKG candidates could be used as reference genes, including seven genes encoding small ribosomal proteins (*rps-23*, *rps-27*, *rps-16*, *rps-26*, *rps-4*, *rps-2*, and *rps-17*), and six genes encoding large ribosomal proteins (*rpl-24.1*, *rpl-15*, *rpl-35*, *rpl-36*, *rpl-27*, and *rpl-33*).

Previous studies suggested that the structure of ribosomes is highly conserved and its composition is constant within a species [69]. Correspondingly, genes encoding ribosomal proteins were considered as housekeeping genes [70]. In *Homo sapiens*, 79 ribosomal and mitoribosomal genes were regarded as housekeeping genes [71,72]. Hence, in this context, 13 housekeeping gene candidates identified in this study are quite credible.

Among 13 HKG candidates, *rps-23* was present in four SGLs, and also widely used as a reference gene for qPCR in many species, such as *Bos indicus* [73], *Homo sapiens* [74], and *Harmonia axyridis* [75]. Additionally, our results strongly support that *rpl-33* and *rps-26* are stable and reliable reference genes in microarray data. Likely, *rps-26* has been widely used as a reference gene to normalize gene expression in *Homo sapiens* [76], *Bovine corpus* [77], *Monotropa hypopitys* [78], and *Millerozyma (Pichia) farinose* [79]. Housekeeping genes *rps-2* and *rps-4* were also identified as stable reference genes in RNA-seq data of *C. elegans*. They were validated and used as suitable references for quantifying gene expressions in *Monotropa hypopitys* [78]. It is worth noting that the most stable gene *rps-23* should be a promising reference gene for both microarray and RNA-seq data. None of the commonly used reference genes in *C. elegans* presented in the top 10 HKG candidates identified in this study. Although some studies confirmed that the orthologous of housekeeping genes identified in this study were reliable and feasible in many species, the utilization of these 13 HKG as reference genes for gene expression quantification in *C. elegans* is quite limited.

It is worth noting that a gene included in at least three second-round ranked gene lists (SGLs) was considered a reliable HKG candidate in this study. Based on this rigorous standard, 13 reliable HKGs were obtained. When we used a more relaxed standard (i.e., the gene included in at least two SGLs is defined as the HKG candidate), another 32 HKGs were also obtained. Among them, several non-ribosomal protein-coding genes, such as *eef-1A.1*, *ubl-1*, *rack-1*, *srh-174*, *ZK721.4*, and *Y116A8C.33*, were included. Some of them were also used as reference genes in some studies where the ribosomal genes would be inappropriate to use as reference genes.

## 5. Conclusions

In this study, 145 microarray datasets generated from 2296 gene array samples of different developmental stages and under various experimental conditions were used to identify stable reference genes for gene expression quantification in *C. elegans*. Based on data normalization, ranking, and integration, our systematic analysis identified and strongly supported 13 HKGs could be used as reliable reference genes, including seven genes encoding small ribosomal proteins (*rps-23*, *rps-27*, *rps-16*, *rps-26*, *rps-4*, *rps-2*, and *rps-17*) and six genes encoding large ribosomal proteins (*rpl-24.1*, *rpl-15*, *rpl-35*, *rpl-36*, *rpl-27* and *rpl-33*). Taken together, our results provide useful guidelines for reference gene(s) selection under different experimental conditions and a possible resource for more accurate and widespread use of qPCR in *C. elegans* and other nematode species.

## Figures and Tables

**Table 1:** Reliable housekeeping gene candidates of *C. elegans* identified in this study.

Gene Symbol	Entrez Gene ID	Description	Chromosome Location	Size (bp)
*rps-23*	178188	40S ribosomal protein S23	Chr. IV: 12390264–12391396	550
*rps-27*	178538	40S ribosomal protein S27	Chr. V: 103394–104064	356
*rps-16*	179998	40S ribosomal protein S16	Chr. V: 15000011–15000594	533
*rps-26*	173342	40S ribosomal protein S26	Chr. I: 14759918–14760654	440
*rps-4*	177481	40S ribosomal protein S4	Chr. IV: 7083694–7084682	849
*rps-2*	177583	40S ribosomal protein S2	Chr. IV: 7925298–7926391	998
*rps-17*	172313	40S ribosomal protein S17	Chr. I: 6220090–6220766	465
*rpl-24.1*	172062	60S ribosomal protein L24	Chr. I: 4585115–4586177	552
*rpl-15*	176891	60S ribosomal protein L15	Chr. IV: 653436–654576	732
*rpl-35*	176097	60S ribosomal protein L35	Chr. III: 7855118–7855680	460
*rpl-36*	176007	60S ribosomal protein L36	Chr. III: 7180249–7180677	355
*rpl-27*	171750	60S ribosomal protein L27	Chr. I: 1834881–1835439	466
*rpl-33*	174166	60S ribosomal protein L35	Chr. II: 7105556–7106462	440

**Table 2:** Validation and comparison for housekeeping gene candidates in *C. elegans*.

Accession Number	Journal, Year	Technique	Sample Size	Data Source	Normalization Method	Top 10 Genes Sorted by *SD* ^1^	Top 10 Genes Sorted by *GC* ^2^
GSE118294	*Eur. J. Cell Biol.*, 2019	DNA Microarray	12	GPL200 Affymetrix *C. elegans* Genome Array	RMA	*rps-26, rpl-36, rpl-33, rpl-15, rps-27, rpl-35, rpl-27, rps-4, rps-23, rpl-24.1*	*rps-26, rpl-33, rpl-36, rps-27, rpl-15, rpl-35, rpl-27, rps-4, rps-23, rpl-24.1*
GSE108968	*Nat. Commun.*, 2019	DNA Microarray	32	GPL10094 Agilent-020186 *C. elegans* (V2) Microarray	Quantile, log	*rpl-24.1, rpl-27, rps-16, rpl-35, cyc-1, rps-23, rpl-33, atp-3, rps-2, rpl-36*	*rpl-33, rpl-24.1, rps-16, rpl-35, rpl-27, rps-23, rpl-36, atp-3, rps-2, cyc-1*
GSE76380	*Cell Metab.*, 2016	DNA Microarray	48	GPL10094 Agilent-020186 *C. elegans* (V2) Microarray	Quantile, log	*rpl-33, rps-2, rps-16, rpl-24.1, rpl-35, rps-23, rps-4, rpl-36, rpl-27, cyc-1*	*rpl-33, rps-23, rps-2, rpl-35, rps-4, rpl-36, rps-16, rpl-15, rpl-24.1, cyc-1*
GSE63528	*eLife*, 2015	RNA-sequencing	36	GPL13657 Illumina HiSeq 2000 (*C. elegans*)	log_2_(CPM+1)	*rps-2, rps-4, pmp-3, atp-3, rbd-1, rpl-27, rps-17, rpl-35, rps-26, act-2*	*rps-4, rps-2, atp-3, rps-26, rps-17, rpl-27, rpl-35, rps-16, pmp-3, rpl-24.1*
GSE60755	*Nature*, 2016	RNA-sequencing	139	GPL13657 Illumina HiSeq 2000 (*C. elegans*)	log_2_(CPM+1)	*rps-2, atp-3, mdh-1, rpl-36, rps-17, rpl-33, rpl-35, rps-4, cyc-1, rpl-15*	*rps-2, atp-3, rpl-36, cyc-1, rpl-35, rpl-33, mdh-1, rps-27, rps-17, rpl-24.1*
GSE98919	*Cell Syst.*, 2017	RNA-sequencing	42	GPL18730 Illumina HiSeq 1500 (*C. elegans*)	log_2_(CPM+1)	*tba-1, rps-4, rpl-27, mdh-1, rpl-15, rps-26, rps-2, rps-23, cyc-1, rpl-24.1*	*rpl-4, rpl-15, rps-23, rpl-27, rpl-24.1, rps-2, rps-26, rps-27, rps-17, rps-16*

^1^*SD* and ^2^ *GC* were abbreviations of standard deviation and Gini coefficient, respectively. Top 10 genes were selected based on the ranking results of the combination gene list of 13 newly identified reference genes (NRGs) and 13 commonly used reference genes (CRGs) in this study.

## Data Availability

Source code for the project is provided at https://github.com/libcell/CE-HKGFinder/under the GNU GPL3.0 License. All data are free to use for non-commercial purpose, and are available at https://drive.google.com/open?id=1zesh6xKr_Hobc6pseqUjC42JtpeGGERq.

## Supplementary Materials

The following are available online at https://www.mdpi.com/2073-4409/9/3/786/s1, Supplementary Material Figure S1: All samples collected in this study; Supplementary Material Figure S2: All published datasets in GPL200 platform used in this study; Supplementary Material Figure S3: Dataset filtering in this study; Supplementary Material Figure S4: Top 5000 genes for first-round ranked gene lists; Supplementary Material Figure S5: Six second-round ranked gene lists; Supplementary Material Figure S6: Result of gene enrichment analysis using Metascape.
