# Supplementary material for: Systematic Identification of Housekeeping Genes Possibly Used as References in Caenorhabditis elegans by Large-Scale Data Integration
Source: Cells. 2020 Mar 24;9(3):786. doi: 10.3390/cells9030786 (PMC7140892; doi:10.3390/cells9030786)
Supplement: Supplementary file 1 [file cells-09-00786-s001.zip › SM/Supplementary Material S2-All published datasets in GPL200 platform.docx]

**Table S1. All published microarray datasets from Affymetrix *C. elegans* Genome Array (GPL200) in GEO database, sorted by release date**

| **No.** | **Accession** | **Title** | **Series type(s)** | ***Organism(s)*** | **Samples** | **Supplementary** | **Release date** |
| --- | --- | --- | --- | --- | --- | --- | --- |
| 1 | GSE1762 | *C. elegans* daf2 mutant adults | Expression profiling by array | *Caenorhabditis elegans* | 20 |  | Sep 14, 2004 |
| 2 | GSE2180 | *C. elegans* embryonic timecourse in wt and mutant embryos | Expression profiling by array | *Caenorhabditis elegans* | 123 | CEL EXP | Apr 01, 2005 |
| 3 | GSE2862 | Twist Heat Shock in *C. elegans* | Expression profiling by array | *Caenorhabditis elegans* | 12 |  | Jan 01, 2006 |
| 4 | GSE2210 | Microarray-assisted Cloning of Mutants: Expression Profiling of tom-1 and unc-43 | Expression profiling by array | *Caenorhabditis elegans* | 9 | CEL | Jan 02, 2006 |
| 5 | GSE6563 | Analysis of expression of genes regulated by DAF-19 | Expression profiling by array | *Caenorhabditis elegans* | 4 | CEL | Dec 21, 2006 |
| 6 | GSE5793 | *C. elegans* gene expression in response to the pathogenic P. aeruginosa strain PA14. | Expression profiling by array | *Caenorhabditis elegans* | 18 | CEL | Feb 08, 2007 |
| 7 | GSE5801 | Genes regulated by PMK-1 and DAF-16 in a daf-2(e1368) background. | Expression profiling by array | *Caenorhabditis elegans* | 9 | CEL | Feb 08, 2007 |
| 8 | GSE7354 | Regulation of presenilin genes (rogae-affy-c.ele-431660) | Expression profiling by array | *Caenorhabditis elegans* | 2 | CEL | Mar 23, 2007 |
| 9 | GSE6547 | Developmental transcriptome profiling of the *C. elegans* pocket protein ortholog, lin-35 | Expression profiling by array | *Caenorhabditis elegans* | 18 | CEL | Apr 27, 2007 |
| 10 | GSE4766 | Decline of Nucleotide Excision Repair Capacity in Aged *Caenorhabditis elegans* | Expression profiling by array | *Caenorhabditis elegans* | 8 | CEL TXT | May 16, 2007 |
| 11 | GSE8159 | A gene expression fingerprint of *C. elegans* embryonic motor neurons. | Expression profiling by array | *Caenorhabditis elegans* | 7 | CEL EXP | Jun 18, 2007 |
| 12 | GSE8004 | Cell-specific microarray profiling of the *C. elegans* nervous system. | Expression profiling by array | *Caenorhabditis elegans* | 24 | CEL CHP EXP | Jun 25, 2007 |
| 13 | GSE8462 | The embryonic muscle transcriptome of *Caenorhabditis elegans* | Expression profiling by array | *Caenorhabditis elegans* | 13 | CEL EXP | Jul 13, 2007 |
| 14 | GSE8659 | Whole genome microarray analysis of *C. elegans* rrf-3 and eri-1 mutants | Expression profiling by array | *Caenorhabditis elegans* | 9 | CEL CHP | Oct 19, 2007 |
| 15 | GSE9301 | SKN-1-dependent oxidative stress response in *C. elegans* | Expression profiling by array | *Caenorhabditis elegans* | 11 | CEL | Nov 01, 2007 |
| 16 | GSE9485 | cRNA amplification methods enhance microarray identification of transcripts expressed in the nervous system | Expression profiling by array | *Caenorhabditis elegans* | 8 | CEL CHP | Nov 01, 2007 |
| 17 | GSE8231 | The embryonic muscle transcriptome of *C. elegans* | Expression profiling by array | *Caenorhabditis elegans* | 12 | CEL | Nov 08, 2007 |
| 18 | GSE9665 | Pairing competitive and topologically distinct regulatory modules enhances patterned gene expression | Expression profiling by array | *Caenorhabditis elegans* | 74 | CEL | Feb 01, 2008 |
| 19 | GSE9896 | Expression data from wildtype and gas-1 mitochondrial mutant *C. elegans* | Expression profiling by array | *Caenorhabditis elegans* | 10 | CEL | Feb 14, 2008 |
| 20 | GSE9897 | Expression data from 2 wildtype and 8 *C. elegans* ETC mutants | Expression profiling by array | *Caenorhabditis elegans* | 10 | CEL | Feb 14, 2008 |
| 21 | GSE9967 | Expression data from wildtype and *C. elegans* mutants | Expression profiling by array | *Caenorhabditis elegans* | 20 | CEL | Feb 14, 2008 |
| 22 | GSE9246 | Transcriptome profiling of slr-2, *C. elegans* C2H2 Zn-finger | Expression profiling by array | *Caenorhabditis elegans* | 6 | CEL | Mar 30, 2008 |
| 23 | GSE8696 | Heme homeostasis is regulated by the conserved and concerted functions of HRG-1 proteins | Expression profiling by array | *Caenorhabditis elegans* | 9 | CEL | Jun 13, 2008 |
| 24 | GSE13258 | RNA interference and retinoblastoma related genes are required for repression of endogenous siRNA targets in *C. elegans* | Expression profiling by array | *Caenorhabditis elegans* | 15 | CEL | Dec 01, 2008 |
| 25 | GSE9682 | Dietary restriction in *Caenorhabditis elegans* | Expression profiling by array | *Caenorhabditis elegans* | 24 | CEL | Dec 15, 2008 |
| 26 | GSE11055 | Temporal expression analysis of *C. elegans* larvae hatching in the presence and absence of food. | Expression profiling by array | *Caenorhabditis elegans* | 18 | CEL CHP EXP TXT XLS | Feb 27, 2009 |
| 27 | GSE14009 | Nutritional control of gene expression during *C. elegans* L1 arrest and recovery | Expression profiling by array  Genome binding/occupancy profiling by high throughput sequencing | *Caenorhabditis elegans* | 32 | CEL CHP EXP | Feb 27, 2009 |
| 28 | GSE14932 | Ecotoxicity of silver nanoparticles on the soil nematode *Caenorhabditis elegans* using functional ecotoxicogenomics | Expression profiling by array | *Caenorhabditis elegans* | 2 | CEL | Feb 28, 2009 |
| 29 | GSE15016 | Ecotoxicogenomic analysis on the soil nematode *Caenorhabditis elegans* exposed to BPA, DEHP and NP | Expression profiling by array | *Caenorhabditis elegans* | 4 | CEL | Feb 28, 2009 |
| 30 | GSE14640 | A condensin-like dosage compensation complex acts at a distance to control expression throughout the genome | Expression profiling by array  Genome binding/occupancy profiling by genome tiling array | *Caenorhabditis elegans* | 42 | CEL GFF PAIR PDF TXT | Mar 01, 2009 |
| 31 | GSE14649 | DCC binding and function (Expression Analysis) | Expression profiling by array | *Caenorhabditis elegans* | 26 | CEL | Mar 01, 2009 |
| 32 | GSE10787 | Low-intensity microwave irradiation does not substantially alter gene expression in late larval and adult *C. elegans* | Expression profiling by array | *Caenorhabditis elegans* | 14 | CEL CHP EXP RPT | Mar 12, 2009 |
| 33 | GSE12298 | *Caenorhabditis elegans* as a Genomic Model for Toxicology | Expression profiling by array | *Caenorhabditis elegans* | 36 | CEL | Jun 03, 2009 |
| 34 | GSE16753 | *C. elegans* fer-1 mutant gene expression profile | Expression profiling by array | *Caenorhabditis elegans* | 12 | CEL | Jun 25, 2009 |
| 35 | GSE16915 | Bio-electrospraying the nematode *Caenorhabditis elegans* | Expression profiling by array | *Caenorhabditis elegans* | 3 | CEL | Jul 02, 2009 |
| 36 | GSE15762 | Comparison of gene expression between wild type (N2) and hlh-30(tm1978) mutant worms | Expression profiling by array | *Caenorhabditis elegans* | 6 | CEL | Jul 24, 2009 |
| 37 | GSE15527 | Regulation of genes affecting body size and innate immunity by the DBL-1/BMP-like pathway in *Caenorhabditis elegans* | Expression profiling by array | *Caenorhabditis elegans* | 11 | CEL | Jul 28, 2009 |
| 38 | GSE15159 | Transcriptomic response of young adult N2, xpa1, and glp1 nematodes 3h after exposure to 50 J/m2 UVC radiation at 25°C | Expression profiling by array | *Caenorhabditis elegans* | 24 | CEL | Oct 19, 2009 |
| 39 | GSE19138 | Comprehensive discovery of endogenous Argonaute binding sites in *C. elegans* | Expression profiling by array | *Caenorhabditis elegans* | 6 | CEL | Nov 24, 2009 |
| 40 | GSE19310 | Expression data from wild type *C. elegans* and 5 osmotic stress resistant mutants exposed to hyper/isotonic environments | Expression profiling by array | *Caenorhabditis elegans* | 30 | CEL CHP | Dec 04, 2009 |
| 41 | GSE18130 | Expression from *C. elegans* L1 animals | Expression profiling by array | *Caenorhabditis elegans* | 9 | CEL CHP | Jan 01, 2010 |
| 42 | GSE18131 | Expression from *C. elegans* L4 animals | Expression profiling by array | *Caenorhabditis elegans* | 9 | CEL CHP | Jan 01, 2010 |
| 43 | GSE18132 | Dynamic O-GlcNAc cycling at promoters of *C. elegans* genes regulating Longevity, Stress, and Immunity | Expression profiling by array  Genome binding/occupancy profiling by genome tiling array  Other | *Caenorhabditis elegans* | 72 | BAR BED CEL CHP | Jan 01, 2010 |
| 44 | GSE19922 | RIP-chip analysis of the *C. elegans* PUF protein FBF | Expression profiling by array | *Caenorhabditis elegans* | 8 | CEL | Jan 27, 2010 |
| 45 | GSE20053 | *C. elegans* gene expression in response to Y. pestis KIM5 infection | Expression profiling by array | *Caenorhabditis elegans* | 6 | CEL CHP | Feb 05, 2010 |
| 46 | GSE16050 | The microRNA miR-124 controls gene expression in the sensory nervous system of *Caenorhabditis elegans* | Expression profiling by array | *Caenorhabditis elegans* | 12 | CEL | Mar 22, 2010 |
| 47 | GSE16405 | Transcriptional changes in the absence of nth-1, xpa-1 and nth-1;xpa-1 | Expression profiling by array | *Caenorhabditis elegans* | 9 | CEL CHP | Apr 12, 2010 |
| 48 | GSE21467 | Loss of *Caenorhabditis elegans* UNG-1 uracil-DNA glycosylase affects apoptosis in response to DNA damaging agents. | Expression profiling by array | *Caenorhabditis elegans* | 6 | CEL | May 26, 2010 |
| 49 | GSE18157 | Gene expression underlying the effects of biotin deficiency in rat liver, worms and yeast | Expression profiling by array | *Caenorhabditis elegans*  *Rattus norvegicus*  *Saccharomyces cerevisiae*  *Schizosaccharomyces pombe* | 17 | CEL CHP JPG RPT | May 31, 2010 |
| 50 | GSE21819 | *Caenorhabditis elegans* infected with Staphylococcus aureus | Expression profiling by array | *Caenorhabditis elegans* | 6 | CEL CHP | Jun 12, 2010 |
| 51 | GSE21747 | Expression data from *Caenorhabditis elegans* exposed to albendazole | Expression profiling by array | *Caenorhabditis elegans* | 6 | CEL | Jun 14, 2010 |
| 52 | GSE21162 | Expression data from the PVD and OLL neurons in *C. elegans* | Expression profiling by array | *Caenorhabditis elegans* | 6 | CEL | Jun 16, 2010 |
| 53 | GSE23528 | Light/dark- and temperature-regulated transcriptional rhythms in adult *Caenorhabditis elegans* | Expression profiling by array | *Caenorhabditis elegans* | 95 | CEL | Sep 01, 2010 |
| 54 | GSE23843 | RIP-chip analysis of the *C. elegans* GLD-2 and RNP-8 protein | Other | *Caenorhabditis elegans* | 21 | CEL | Sep 21, 2010 |
| 55 | GSE21376 | Developmental Roles of MEC and NuRD Complexes in *Caenorhabditis elegans*. | Expression profiling by array | *Caenorhabditis elegans* | 9 | CEL | Oct 31, 2010 |
| 56 | GSE25513 | AMPK and calcineurin induced longevity is mediated by CRTC-1 and CREB | Expression profiling by array | *Caenorhabditis elegans* | 12 | CEL | Nov 20, 2010 |
| 57 | GSE19972 | Expression Data from Ectopic expression of SUMO-1 in *C. elegans* | Expression profiling by array | *Caenorhabditis elegans* | 9 | CEL CHP | Jan 31, 2011 |
| 58 | GSE21591 | RNA immunoprecipitation of GLD-1 followed by microarray analysis of the co-IP'ed mRNAs | Other  Expression profiling by array | *Caenorhabditis elegans* | 15 | CEL | Feb 04, 2011 |
| 59 | GSE25831 | Fed L1 larvae total RNA levels by microarray | Expression profiling by array | *Caenorhabditis elegans* | 6 | CEL CHP | Mar 01, 2011 |
| 60 | GSE25834 | An MLL/COMPASS subunit functions in the *C. elegans* dosage compensation complex to target X chromosomes for transcriptional regulation of gene expression | Expression profiling by array  Genome binding/occupancy profiling by genome tiling array | *Caenorhabditis elegans* | 53 | CEL CHP GFF PAIR | Mar 01, 2011 |
| 61 | GSE22660 | Expression data from *Caenorhabditis elegans* exposed to ivermectin | Expression profiling by array | *Caenorhabditis elegans* | 20 | CEL | Apr 01, 2011 |
| 62 | GSE28494 | Germline and embryo gene expression of wild-type vs. mutants in lin-54, a component of the *C. elegans* DRM complex | Expression profiling by array | *Caenorhabditis elegans* | 12 | CEL CHP TXT | Apr 09, 2011 |
| 63 | GSE27867 | Expression data from *C. elegans* (wild type vs. tag-24) | Expression profiling by array | *Caenorhabditis elegans* | 6 | CEL | Apr 21, 2011 |
| 64 | GSE28853 | Chromosome-biased binding and gene regulation by the *C. elegans* DRM complex | Genome binding/occupancy profiling by genome tiling array  Expression profiling by array | *Caenorhabditis elegans* | 18 | CEL CHP TXT | Apr 27, 2011 |
| 65 | GSE21784 | Genome-wide expression analysis during aging in *C. elegans* | Expression profiling by array | *Caenorhabditis elegans* | 9 | CEL | May 11, 2011 |
| 66 | GSE27401 | *Caenorhabditis elegans* infection with Candida albicans | Expression profiling by array | *Caenorhabditis elegans* | 9 | CEL TXT | Jun 04, 2011 |
| 67 | GSE28856 | Expression data of wild-type *C. elegans* shifted from 23 degrees to 17 degrees | Expression profiling by array | *Caenorhabditis elegans* | 12 | CEL | Jun 23, 2011 |
| 68 | GSE30505 | Members of the H3K4 trimethylation complex regulate lifespan in a germline-dependent manner in *C. elegans* | Expression profiling by array | *Caenorhabditis elegans* | 23 | CEL | Jul 08, 2011 |
| 69 | GSE25633 | Transcriptional profiling of *C. elegans* DAF-19 uncovers a ciliary base-associated protein and a CDK/CCRK/LF2p-related kinase required for intraflagellar transport. | Expression profiling by array | *Caenorhabditis elegans* | 12 | CEL | Jul 26, 2011 |
| 70 | GSE32339 | Comparison between *Caenorhabditis elegans* wild-type and Nep-1 mutant strains | Expression profiling by array | *Caenorhabditis elegans* | 10 | CEL TXT XLS | Sep 24, 2011 |
| 71 | GSE32949 | Expression data from *C. elegans* | Expression profiling by array | *Caenorhabditis elegans* | 6 | CEL CHP | Oct 14, 2011 |
| 72 | GSE34026 | Expression data from *C. elegans* | Expression profiling by array | *Caenorhabditis elegans* | 15 | CEL CHP | Dec 02, 2011 |
| 73 | GSE23013 | mRNA microarray analysis on young adult *Caenorhabditis elegans* exposed to benzene, toluene, formaldehyde and a BTF mix | Expression profiling by array | *Caenorhabditis elegans* | 5 | CEL | Dec 31, 2011 |
| 74 | GSE24847 | mRNA microarray analysis on young adult *Caenorhabditis elegans* exposed to MWCNT | Expression profiling by array | *Caenorhabditis elegans* | 3 | CEL | Dec 31, 2011 |
| 75 | GSE24923 | mRNA microarray analysis on young adult *Caenorhabditis elegans*, Drosophila melanogaster, and Danio rerio exposed to benzene, toluene, and formaldehyde | Expression profiling by array | *Caenorhabditis elegans*  *Danio rerio*  *Drosophila melanogaster* | 14 | CEL | Dec 31, 2011 |
| 76 | GSE35354 | Meta-analysis of global transcriptomics of Quercetin and Tannic acid exposed *C. elegans* | Expression profiling by array | *Caenorhabditis elegans* | 24 | CEL | Feb 03, 2012 |
| 77 | GSE35360 | The complex interplay of genetic pathways in C.elegans following the treatment with humic substances | Expression profiling by array | *Caenorhabditis elegans* | 27 | CEL | Feb 03, 2012 |
| 78 | GSE34113 | Expression Profiling of Caenorabditis elegans tdp-1 loss-of-function mutant | Expression profiling by array | *Caenorhabditis elegans* | 6 | CEL | Feb 21, 2012 |
| 79 | GSE32942 | Microarray analysis of TAP::ALG-1 associated RNAs isolated from synchronized 'wild-type' animals and 'mir-58' mutants | Expression profiling by array | *Caenorhabditis elegans* | 6 | CEL | Mar 01, 2012 |
| 80 | GSE32944 | Identification of miRNA target genes in *C. elegans* by RIP-chip-SRM | Expression profiling by array  Other | *Caenorhabditis elegans* | 13 | CEL GPR | Mar 01, 2012 |
| 81 | GSE36358 | Expression Data from International C.elegans Experiment 1st | Expression profiling by array | *Caenorhabditis elegans* | 5 | CEL | Mar 31, 2012 |
| 82 | GSE37266 | Stimulation of Host Immune Defenses by a Small Molecule Protects *C. elegans* from Bacterial Infection | Expression profiling by array | *Caenorhabditis elegans* | 6 | CEL | Apr 14, 2012 |
| 83 | GSE32521 | Toxicogenomic responses of *C. elegans* to gold nanoparticles | Expression profiling by array | *Caenorhabditis elegans* | 6 | CEL | Apr 16, 2012 |
| 84 | GSE37432 | Function, targets and evolution of *Caenorhabditis elegans* piRNAs (mRNA) | Expression profiling by array | *Caenorhabditis elegans* | 9 | CEL | Jun 18, 2012 |
| 85 | GSE37433 | Function, targets and evolution of *Caenorhabditis elegans* piRNAs | Expression profiling by array  Non-coding RNA profiling by high throughput sequencing | *Caenorhabditis elegans* | 16 | BED CEL | Jun 18, 2012 |
| 86 | GSE39012 | Expression data from L3 stage Caernorhabditis elegans after arsenic exposure | Expression profiling by array | *Caenorhabditis elegans* | 9 | CEL CHP | Jun 29, 2012 |
| 87 | GSE38196 | ATFS-1 mediates a protective transcription program during mitochondrial stress | Expression profiling by array | *Caenorhabditis elegans* | 12 | CEL CHP | Jul 12, 2012 |
| 88 | GSE32031 | Expression data in *C. elegans* L2 larvae after nhr-23 inhibition and in controls | Expression profiling by array | *Caenorhabditis elegans* | 6 | CEL | Aug 29, 2012 |
| 89 | GSE32467 | Expression data from wildtype and unc-37 mutant A-class motor neurons in *C. elegans* | Expression profiling by array | *Caenorhabditis elegans* | 6 | CEL | Sep 01, 2012 |
| 90 | GSE38997 | Expression data from *Caenorhabditis elegans* L1-stage larvae exposed to ethidium bromide and/or serial ultraviolet C radiation | Expression profiling by array | *Caenorhabditis elegans* | 69 | CEL | Oct 01, 2012 |
| 91 | GSE41486 | Expression data from *Caenorhabditis elegans* exposed to single-walled carbon nanotubes | Expression profiling by array | *Caenorhabditis elegans* | 2 | CEL CHP | Oct 11, 2012 |
| 92 | GSE42592 | Alterations in gene expression in *Caenorhabditis elegans* associated with exposure to several classes of toxic industrial chemicals/materials. | Expression profiling by array | *Caenorhabditis elegans* | 80 | CEL CHP | Nov 28, 2012 |
| 93 | GSE42703 | Expression data from *C. elegans* in the presence or absence of copper sulfate | Expression profiling by array | *Caenorhabditis elegans* | 4 | CEL CHP | Dec 05, 2012 |
| 94 | GSE42192 | Gene expression data from C.elegans | Expression profiling by array | *Caenorhabditis elegans* | 18 | CEL | Dec 31, 2012 |
| 95 | GSE34471 | Heme utilization in the *Caenorhabditis elegans* hypodermal cells is facilitated by HRG-2 | Expression profiling by array | *Caenorhabditis elegans* | 12 | CEL CHP | Jan 01, 2013 |
| 96 | GSE27677 | A conserved JNK/AP-1 module is a key mediator of intermittent fasting-induced longevity in *C. elegans* | Expression profiling by array | *Caenorhabditis elegans* | 46 | CEL | Mar 15, 2013 |
| 97 | GSE43952 | Integration of Metabolic and Gene Regulatory Networks Modulates The *C. elegans* Dietary Response | Expression profiling by array | *Caenorhabditis elegans* | 9 | CEL | Mar 28, 2013 |
| 98 | GSE43953 | Diet specific expression profiles_MA2 | Expression profiling by array | *Caenorhabditis elegans* | 9 | CEL | Mar 28, 2013 |
| 99 | GSE43954 | Diet specific expression profiles_MA1 | Expression profiling by array | *Caenorhabditis elegans* | 9 | CEL | Mar 28, 2013 |
| 100 | GSE43959 | Diet-Induced Developmental Acceleration Independent of TOR and Insulin in *C. elegans* | Expression profiling by array | *Caenorhabditis elegans* | 18 | CEL | Mar 28, 2013 |
| 101 | GSE42689 | mek-1 and mlk-1 in fed or fasted condition | Expression profiling by array | *Caenorhabditis elegans* | 10 | CEL | Apr 22, 2013 |
| 102 | GSE39252 | Expression changes in *Caenorhabditis elegans* xpa-1 mutant | Expression profiling by array | *Caenorhabditis elegans* | 10 | CEL | May 03, 2013 |
| 103 | GSE41056 | Analysis of gene expression changes upon infection of C.elegans with Orsay virus | Expression profiling by array | *Caenorhabditis elegans* | 20 | CEL | Jun 01, 2013 |
| 104 | GSE41058 | Competition between viral-derived and endogenous small RNA pathways regulates gene expression in response to viral infection in C.elegans. | Expression profiling by array   - Non-coding RNA profiling by high throughput sequencing | *Caenorhabditis elegans* | 26 | CEL FA | Jun 01, 2013 |
| 105 | GSE16975 | Hydrogen sulfide (H2S) modulates lifespan of C.elegans | Expression profiling by array | *Caenorhabditis elegans* | 6 | CEL | Jul 30, 2013 |
| 106 | GSE40127 | GEI-8, a homologue of vertebrate nuclear receptor corepressor NCoR/SMRT, regulates development and neuronal functions in *C. elegans*. | Expression profiling by array | *Caenorhabditis elegans* | 6 | CEL CHP | Aug 11, 2013 |
| 107 | GSE50513 | Identify genes regulated by zip-2 in absence and presence of P. aeruginosa PA14 infection at 4h | Expression profiling by array | *Caenorhabditis elegans* | 12 | CEL TXT | Aug 30, 2013 |
| 108 | GSE48347 | *C. elegans* mixed stage embryo total RNA levels by microarray: L4440 RNAi, smo-1 RNAi and sdc-2 (y93)+RNAi | Expression profiling by array | *Caenorhabditis elegans* | 9 | CEL CHP | Aug 31, 2013 |
| 109 | GSE39145 | Multiple DNA repair pathways collectively protect against DNA damage-induced replicative aging. | Expression profiling by array | *Caenorhabditis elegans* | 8 | CEL | Sep 15, 2013 |
| 110 | GSE41366 | Alterations in gene expression in *Caenorhabditis elegans* associated with organophosphate pesticide intoxication and recovery | Expression profiling by array | *Caenorhabditis elegans* | 146 | CEL | Oct 05, 2013 |
| 111 | GSE51691 | Expression data from glp-1(e2141) hermaphrodites maintained with or without males for 8 days | Expression profiling by array | *Caenorhabditis elegans* | 11 | CEL | Oct 25, 2013 |
| 112 | GSE52064 | DRM complex mutant lin-54 vs. H3K36 methyltransferase mutant mes-4 vs. lin-54; mes-4 double mutant vs. wild type C.elegans germline | Expression profiling by array | *Caenorhabditis elegans* | 12 | CEL | Nov 05, 2013 |
| 113 | GSE44318 | Expression data from *Caenorhabditis elegans* fed with 13L cocoa peptide | Expression profiling by array | *Caenorhabditis elegans* | 7 | CEL | Dec 31, 2013 |
| 114 | GSE46288 | Expression data from *C. elegans* during L3 and L3-lethargus | Expression profiling by array | *Caenorhabditis elegans* | 6 | CEL | Apr 18, 2014 |
| 115 | GSE46289 | Expression data from *C. elegans* during L4, L4-lethargus, and Adult | Expression profiling by array | *Caenorhabditis elegans* | 15 | CEL | Apr 18, 2014 |
| 116 | GSE46291 | *C. elegans* during lethargus | Expression profiling by array | *Caenorhabditis elegans* | 21 | CEL | Apr 18, 2014 |
| 117 | GSE54024 | Expression Data of Reactive Oxygen Species Signalling in *C. elegans* | Expression profiling by array | *Caenorhabditis elegans* | 22 | CEL | May 08, 2014 |
| 118 | GSE51502 | Use of an activated beta-catenin to identify Wnt/beta-catenin pathway target genes in *C. elegans*, including a subset of collagen genes expressed in late larval development | Expression profiling by array | *Caenorhabditis elegans* | 6 |  | May 13, 2014 |
| 119 | GSE45651 | Expression data from the starved first larval stage (L1) *C. elegans* animals that were incubated in S-basal buffer for 30 hours after bleaching | Expression profiling by array | *Caenorhabditis elegans* | 6 | CEL | May 16, 2014 |
| 120 | GSE29951 | Drivers of metallothionein-mediated detoxification of cadmium in C.elegans | Expression profiling by array | *Caenorhabditis elegans* | 12 | CEL | Jun 01, 2014 |
| 121 | GSE54011 | *C. elegans* expression: toxic vs. adequate vs. low selenium | Expression profiling by array | *Caenorhabditis elegans* | 5 | CEL TXT | Jul 03, 2014 |
| 122 | GSE54513 | mRNAs that co-purify with OMA-1 in the *C. elegans* germline (microarray) | Expression profiling by array | *Caenorhabditis elegans* | 12 | CEL | Sep 29, 2014 |
| 123 | GSE54518 | mRNAs that co-purify with OMA-1 in the *C. elegans* germline | Expression profiling by array  Other | *Caenorhabditis elegans* | 13 | CEL TXT | Sep 29, 2014 |
| 124 | GSE57664 | Gene Expression Profiling Reveals Molecular Patterns Underlying the Lifespan-Extending Effect of Tyrosol in *Caenorhabditis elegans* | Expression profiling by array | *Caenorhabditis elegans* | 6 | CEL | Nov 01, 2014 |
| 125 | GSE47778 | DAF-16/FoxO and EGL-27/GATA promote developmental growth in response to persistent somatic DNA damage | Expression profiling by array | *Caenorhabditis elegans* | 51 | CEL | Nov 18, 2014 |
| 126 | GSE51161 | DAF-16/FoxO and EGL-27/GATA promote developmental growth in response to persistent somatic DNA damage [N2, xpa-1] | Expression profiling by array | *Caenorhabditis elegans* | 15 | CEL | Nov 18, 2014 |
| 127 | GSE51162 | DAF-16/FoxO and EGL-27/GATA promote developmental growth in response to persistent somatic DNA damage [N2, daf-2, daf-16, daf-2;daf-16] | Expression profiling by array | *Caenorhabditis elegans* | 36 | CEL | Nov 18, 2014 |
| 128 | GSE45292 | In vivo gene expression analysis of C elegans in response to rifampicin | Expression profiling by array | *Caenorhabditis elegans* | 4 | CEL | Dec 31, 2014 |
| 129 | GSE65851 | Beta Amyloid toxicity in a *Caenorhabditis elegans* model of Alzheimer's disease | Expression profiling by array | *Caenorhabditis elegans* | 47 | CEL CHP | Feb 12, 2015 |
| 130 | GSE53732 | Conserved nutrient sensor O-GlcNAc transferase is integral to the *C. elegans* pathogen-specific immune response | Expression profiling by array | *Caenorhabditis elegans* | 36 | CEL | May 01, 2015 |
| 131 | GSE68709 | Exposure of *C. elegans* to AOBr-containing surface water samples and to a M. aeruginosa batch culture | Expression profiling by array | *Caenorhabditis elegans* | 15 | CEL | May 12, 2015 |
| 132 | GSE71618 | Expression data from DD neurons isolated from early L1 stage *C. elegans* larvae. | Expression profiling by array | *Caenorhabditis elegans* | 7 | CEL XLSX | Aug 01, 2015 |
| 133 | GSE70509 | Toxicogenomic responses of *Caenorhabditis elegans* exposed to sulfidized silver nanoparticles | Expression profiling by array | *Caenorhabditis elegans* | 12 | CEL CHP | Aug 22, 2015 |
| 134 | GSE70693 | Comparison of gene expression between sams-1(RNAi) and sams-1(RNAi) animals rescued by choline | Expression profiling by array | *Caenorhabditis elegans* | 12 | CEL | Aug 27, 2015 |
| 135 | GSE72029 | *C. elegans* gene expression in healthy and PA14-infected wild-type and fshr-1 mutant worms | Expression profiling by array | *Caenorhabditis elegans* | 12 | CEL CHP | Sep 30, 2015 |
| 136 | GSE52747 | Transcriptional response induced by Wnt signaling in *Caenorhabditis elegans* affects lateral and ventral hypodermal cell development | Expression profiling by array | *Caenorhabditis elegans* | 9 | CEL | Nov 18, 2015 |
| 137 | GSE63531 | Expression data from *Caenorhabditis elegans* fed with the bacterial strain Bifidobacterium animalis sbsp. lactis CECT 8145 | Expression profiling by array | *Caenorhabditis elegans* | 8 | CEL | Nov 22, 2015 |
| 138 | GSE73070 | RIP-Chip analysis of the *C. elegans* FOG-1 and FOG-3 proteins | Expression profiling by array | *Caenorhabditis elegans* | 40 | CEL | Dec 14, 2015 |
| 139 | GSE64336 | Expression data of worms under different caloric restriction mimetic treatments | Expression profiling by array | *Caenorhabditis elegans* | 15 | CEL | Dec 22, 2015 |
| 140 | GSE65417 | Expression data from *C. elegans* wild type, hlh-25 mutant and hlh-29 mutant strains | Expression profiling by array | *Caenorhabditis elegans* | 9 | CEL CHP RPT TXT | Jan 01, 2016 |
| 141 | GSE74459 | Translation State Array Assay for C elegans IFE-1-dependent mRNAs | Expression profiling by array | *Caenorhabditis elegans* | 12 | CEL XLS XLSX | Jan 07, 2016 |
| 142 | GSE64973 | Analysis of gene expression between monomethyl branched-chain fatty acid deficient (elo-5) and wild type N2 *C. elegans* L1 larvae hatched on food-free NGM plates | Expression profiling by array | *Caenorhabditis elegans* | 6 | CEL DCP | Jan 14, 2016 |
| 143 | GSE77109 | C.elegans gene expression study for single, double or triple genetic perturbations of regulators in AMPK, Insulin and TOR pathway | Expression profiling by array | *Caenorhabditis elegans* | 20 | CEL | Jan 22, 2016 |
| 144 | GSE77110 | C.elegans time course study on dietary restriction and aging | Expression profiling by array | *Caenorhabditis elegans* | 15 | CEL | Jan 22, 2016 |
| 145 | GSE77111 | A Systems Approach to Reverse Engineer Lifespan Extension by Dietary Restriction | Expression profiling by array | *Caenorhabditis elegans* | 35 | CEL | Jan 22, 2016 |
| 146 | GSE73669 | ATFS-1 regulates a broad protective transcriptional program | Expression profiling by array | *Caenorhabditis elegans* | 6 | CEL CHP | Mar 21, 2016 |
| 147 | GSE84441 | mRNA microarray analysis on young adults C.elegans exposed to UV, TiO2 and UV+TiO2 | Expression profiling by array | *Caenorhabditis elegans* | 12 | CEL | Jul 16, 2016 |
| 148 | GSE84489 | mRNA microarray analysis on young adults C.elegans exposed to GO and rGO | Expression profiling by array | *Caenorhabditis elegans* | 3 | CEL | Jul 19, 2016 |
| 149 | GSE85342 | A High-Content, Phenotypic Screen Identifies Fluorouridine as an Inhibitor of Pyoverdine Biosynthesis and Pseudomonas aeruginosa Virulence | Expression profiling by array | *Caenorhabditis elegans* | 6 | CEL | Aug 10, 2016 |
| 150 | GSE83722 | Lipid biosynthesis coordinates a Mitochondrial to Cytosolic Stress Response | Expression profiling by array | *Caenorhabditis elegans* | 12 | CEL | Sep 08, 2016 |
| 151 | GSE87052 | NIPI-3 regulates the expression of *C. elegans* immune genes | Expression profiling by array | *Caenorhabditis elegans* | 9 | CEL | Sep 18, 2016 |
| 152 | GSE94701 | Expression data from mir-35-41(nDf50) mutant embryos grown at 20 degrees, compared to wild type | Expression profiling by array | *Caenorhabditis elegans* | 6 | CEL TXT | Feb 09, 2017 |
| 153 | GSE94702 | Expression data from mir-35-41(nDf50) mutant embryos grown at 25 degrees, compared to wild type | Expression profiling by array | *Caenorhabditis elegans* | 6 | CEL TXT | Feb 09, 2017 |
| 154 | GSE94704 | A microRNA family exerts maternal control on sex determination in *C. elegans* | Expression profiling by array  Expression profiling by high throughput sequencing | *Caenorhabditis elegans* | 16 | CEL | Feb 09, 2017 |
| 155 | GSE95603 | Expression data from *C. elegans* treated with anatase TiO2 nanoparticles | Expression profiling by array | *Caenorhabditis elegans* | 2 | CEL | Mar 03, 2017 |
| 156 | GSE84894 | Expression data from starved first larval stage of wildtype and hyl-1(ok976); lagr-1(gk327) *C. elegans* | Expression profiling by array | *Caenorhabditis elegans* | 6 | CEL | Mar 07, 2017 |
| 157 | GSE89731 | Octopamine Enhances Oxidative Stress Resistance Through the Fasting-Responsive Transcription Factor DAF-16/FOXO in *C. elegans* | Expression profiling by array | *Caenorhabditis elegans* | 6 | CEL | Mar 08, 2017 |
| 158 | GSE51046 | Cholesterol regulates DAF-16 nuclear localization and fasting-induced longevity in *C. elegans* | Expression profiling by array | *Caenorhabditis elegans* | 8 | CEL | May 01, 2017 |
| 159 | GSE81854 | Grape seed extracts-mediated lipid mobility in *C. elegans* | Expression profiling by array | *Caenorhabditis elegans* | 2 | CEL | May 24, 2017 |
| 160 | GSE99201 | Wild-type and daf-16(mgDf50) L1 larvae 3 hours after hatching in the presence or absence of food | Expression profiling by array | *Caenorhabditis elegans* | 12 | CEL CSV TXT | May 24, 2017 |
| 161 | GSE71482 | Expression data from *Caenorhabditis elegans* fed with a Lactoferrin-based product | Expression profiling by array | *Caenorhabditis elegans* | 8 | CEL TXT | Jun 12, 2017 |
| 162 | GSE55422 | A Conserved Mitochondrial Surveillance Pathway Is Required for Defense against Pseudomonas aeruginosa | Expression profiling by array | *Caenorhabditis elegans* | 21 | CEL | Jun 20, 2017 |
| 163 | GSE100814 | Tocotrienol Rich Fraction (TRF) Modulates Genes Expression in Oxidative Stress-Induced *Caenorhabditis elegans* | Expression profiling by array | *Caenorhabditis elegans* | 18 | CEL CHP | Jul 06, 2017 |
| 164 | GSE89614 | Role of the MicroRNA Machinery in Fasting-Induced Gene Expression Changes and Longevity in *C. elegans* (mRNA) | Expression profiling by array | *Caenorhabditis elegans* | 8 | CEL | Aug 30, 2017 |
| 165 | GSE89624 | Role of the MicroRNA Machinery in Fasting-Induced Gene Expression Changes and Longevity in *C. elegans* | Expression profiling by array  Non-coding RNA profiling by array | *Caenorhabditis elegans*  *synthetic construct* | 11 | CEL | Aug 30, 2017 |
| 166 | GSE37303 | Fasting induced changes in gene expression profiles of *C. elegans* | Expression profiling by array | *Caenorhabditis elegans* | 12 | CEL | Dec 07, 2017 |
| 167 | GSE37305 | Age and Fasting in *C. elegans* | Expression profiling by array  Expression profiling by genome tiling array | *Caenorhabditis elegans* | 12 | BAR CEL TXT | Dec 08, 2017 |
| 168 | GSE96068 | The RFX transcription factor gene, daf-19, has dual funcitons in ciliated and non-ciliated neurons | Expression profiling by array | *Caenorhabditis elegans* | 14 | CEL | Jan 31, 2018 |
| 169 | GSE95636 | *Caenorhabditis elegans* infected with Enterococcus | Expression profiling by array | *Caenorhabditis elegans* | 13 | CEL | Feb 14, 2018 |
| 170 | GSE40371 | Expression from *C. elegans* L1 animals | Expression profiling by array | *Caenorhabditis elegans* | 9 | CEL CHP | Jun 04, 2018 |
| 171 | GSE48499 | Transcriptional difference between wild-type and zip-3(gk3164) | Expression profiling by array | *Caenorhabditis elegans* | 6 | CEL CHP | Nov 01, 2018 |

Notes: the rows with background painted by pale orange, light grey and apple green colors represented the 18 SuperSeries, 5 smaller datasets and 3 studies without raw data, respectively. And, these datasets were excluded in subsequent analysis. In total, 145 independent microarray datasets were adopted in this study.
