# Supplementary material for: Systematic Identification of Housekeeping Genes Possibly Used as References in Caenorhabditis elegans by Large-Scale Data Integration
Source: Cells. 2020 Mar 24;9(3):786. doi: 10.3390/cells9030786 (PMC7140892; doi:10.3390/cells9030786)
Supplement: Supplementary file 1 [file cells-09-00786-s001.zip › SM/Supplementary Material S3-dataset filtering in this study.docx]

**Table S2. The relationships between SuperSeries and SubSeries used in this study**

| **SuperSeries** | **SubSeries** | **Platform(s)** | **Remaining?** |
| --- | --- | --- | --- |
| GSE18132 | GSE18130 | GPL200 [Celegans] Affymetrix C. elegans Genome Array | yes |
|  | GSE18131 | GPL200 [Celegans] Affymetrix C. elegans Genome Array | yes |
|  | GSE18611 | GPL5634 [Ce25b_MR] Affymetrix C. elegans Tiling 1.0R Array | no |
|  | GSE40371 | GPL200 [Celegans] Affymetrix C. elegans Genome Array | yes |
| GSE37305 | GSE37303 | GPL200 [Celegans] Affymetrix C. elegans Genome Array | yes |
| GSE89624 | GSE89609 | GPL21572 [miRNA-4] Affymetrix Multispecies miRNA-4 Array [ProbeSet ID version] | no |
|  | GSE89614 | GPL200 [Celegans] Affymetrix C. elegans Genome Array | yes |
| GSE94704 | GSE94701 | GPL200 [Celegans] Affymetrix C. elegans Genome Array | yes |
|  | GSE94702 | GPL200 [Celegans] Affymetrix C. elegans Genome Array | yes |
|  | GSE94703 | GPL19757 Illumina NextSeq 500 (Caenorhabditis elegans) | no |
| GSE77111 | GSE77109 | GPL200 [Celegans] Affymetrix C. elegans Genome Array | yes |
|  | GSE77110 | GPL200 [Celegans] Affymetrix C. elegans Genome Array | yes |
| GSE47778 | GSE51161 | GPL200 [Celegans] Affymetrix C. elegans Genome Array | yes |
|  | GSE51162 | GPL200 [Celegans] Affymetrix C. elegans Genome Array | yes |
| GSE43959 | GSE43953 | GPL200 [Celegans] Affymetrix C. elegans Genome Array | yes |
|  | GSE43954 | GPL200 [Celegans] Affymetrix C. elegans Genome Array | yes |
| GSE9967 | GSE9896 | GPL200 [Celegans] Affymetrix C. elegans Genome Array | yes |
|  | GSE9897 | GPL200 [Celegans] Affymetrix C. elegans Genome Array | yes |
| GSE54518 | GSE54513 | GPL200 [Celegans] Affymetrix C. elegans Genome Array | yes |
|  | GSE54517 | GPL13657 Illumina HiSeq 2000 (Caenorhabditis elegans) | no |
| GSE46291 | GSE46288 | GPL200 [Celegans] Affymetrix C. elegans Genome Array | yes |
|  | GSE46289 | GPL200 [Celegans] Affymetrix C. elegans Genome Array | yes |
| GSE41058 | GSE41056 | GPL200 [Celegans] Affymetrix C. elegans Genome Array | yes |
|  | GSE41057 | GPL13657 Illumina HiSeq 2000 (Caenorhabditis elegans) | no |
| GSE37433 | GSE28617 | GPL9269 Illumina Genome Analyzer II (Caenorhabditis elegans) | no |
|  | GSE37432 | GPL200 [Celegans] Affymetrix C. elegans Genome Array | yes |
| GSE32944 | GSE32941 | GPL14724 Washington University/Genome Sequencing Center C. elegans 23K [oligoID version] | no |
|  | GSE32942 | GPL200 [Celegans] Affymetrix C. elegans Genome Array | yes |
|  | GSE32943 | GPL14724 Washington University/Genome Sequencing Center C. elegans 23K [oligoID version] | no |
| GSE24923 | GSE23013 | GPL200 [Celegans] Affymetrix C. elegans Genome Array | yes |
|  | GSE24845 | GPL1322 [Drosophila_2] Affymetrix Drosophila Genome 2.0 Array | no |
|  | GSE24846 | GPL1319 [Zebrafish] Affymetrix Zebrafish Genome Array | no |
| GSE28853 | GSE28494 | GPL200 [Celegans] Affymetrix C. elegans Genome Array | yes |
|  | GSE28852 | GPL7482 C. elegans 385K ChIP01-3 | no |
| GSE25834 | GSE25831 | GPL200 [Celegans] Affymetrix C. elegans Genome Array | yes |
|  | GSE25833 | GPL8134 NimbleGen 071121_Celegans180_ChIP03_design_ID_6737 | no |
|  | GSE25877 | GPL8134 NimbleGen 071121_Celegans180_ChIP03_design_ID_6737 | no |
| GSE14640 | GSE14649 | GPL200 [Celegans] Affymetrix C. elegans Genome Array | yes |
|  | GSE14650 | GPL8133 NimbleGen 2006-05-26_CE_WS158_ChIP design_ID 3801 | no |
|  | GSE14651 | GPL7098 NimbleGen C_elegans_WS170_Tiling_Iso_HX1 tiling design | no |
|  | GSE14652 | GPL8134 NimbleGen 071121_Celegans180_ChIP03_design_ID_6737 | no |
|  | GSE14653 | GPL8135 NimbleGen 080319_C_elegans_ChIP_03_design_ID-7348 | no |
| GSE14009 | GSE11055 | GPL200 [Celegans] Affymetrix C. elegans Genome Array | yes |
|  | GSE13973 | GPL9269 Illumina Genome Analyzer II (Caenorhabditis elegans) | no |

**Notes:** Up to the end of 2018, 171 GEO Series were published in GEO database, with microarray GPL200 platform. Of which, 18 superSeries were removed as shown in above table. GSE81854, GSE95603, GSE41486, GSE7354 and GSE14932 were removed because of small sample size (less than 3). In addition, GSE51502, GSE2862 and GSE1762 were also removed because their raw data were not supplied. Additionally, though several samples were included in GSE6547 and GSE9246, GSE15016 and GSE14932 simultaneously, the first three datasets were remained because of few repeated samples among these four datasets.
