# Supplementary material for: Systematic Identification of Housekeeping Genes Possibly Used as References in Caenorhabditis elegans by Large-Scale Data Integration
Source: Cells. 2020 Mar 24;9(3):786. doi: 10.3390/cells9030786 (PMC7140892; doi:10.3390/cells9030786)
Supplement: Supplementary file 1 [file cells-09-00786-s001.zip › SM/Supplementary Material S5-Second-round ranked genes (top 50, six sets).docx]

**Table S3. Housekeeping gene candidates from six sets, in *C. elegans***

| **Rank** | **SGL_R_** | **SGL_M_** | **SGL_G_** | **SGL_L_** | **SGL_P_** | **SGL_V_** |
| --- | --- | --- | --- | --- | --- | --- |
| 1 | *rps-26* | *sar-1* | *6r55.2* | *rps-26* | *rps-1* | *sydn-1* |
| 2 | *fbxa-118* | *cap-1* | *f32b4.6* | *rpl-24.1* | *rps-27* | *T20F7.5* |
| 3 | *rps-27* | *arf-3* | *abt-6* | *rps-27* | *rpl-17* | *F16B12.1* |
| 4 | *rpl-24.1* | *pdcd-2* | *abch-1* | *rps-11* | *rpl-23* | *M151.1* |
| 5 | *rpl-16* | *idhg-1* | *aat-7* | *rpl-35* | *rps-4* | *lgc-33* |
| 6 | *rpl-17* | *pfkb-1.1* | *abu-2* | *rps-0* | *eef-1A.1* | *jip-1* |
| 7 | *rps-16* | *coq-6* | *ac7.3* | *glr-2* | *rpl-3* | *let-765* |
| 8 | *rps-1* | *cey-4* | *acr-5* | *rps-16* | *e03h4.2* | *amt-3* |
| 9 | *rpl-3* | *fce-1* | *abf-3* | *rpl-19* | *rps-2* | *srt-23* |
| 10 | *rpl-27* | *let-70* | *abf-4* | *rpl-10* | *rpl-36* | *Y116A8C.33* |
| 11 | *nhr-174* | *Y57G11C.1147* | *acp-3* | *rpl-16* | *ubl-1* | *Y53G8AM.8* |
| 12 | *rps-14* | *dlc-1* | *acd-4* | *rpl-13* | *rpl-25.2* | *gpx-8* |
| 13 | *rps-3* | *eef-1G* | *adpr-1* | *rpl-15* | *rps-21* | *Y67D8A.2* |
| 14 | *ubl-1* | *kin-3* | *acdh-5* | *rps-23* | *rpl-30* | *gcy-29* |
| 15 | *rpl-33* | *ubq-1* | *acc-1* | *ZK721.4* | *c37a2.7* | *Y92H12A.5* |
| 16 | *rps-2* | *pqn-53* | *acr-20* | *col-93* | *y37e3.8* | *clec-135* |
| 17 | *rpl-23* | *k12h4.4* | *acc-2* | *rpl-36* | *rpl-33* | *hpo-11* |
| 18 | *rpl-10* | *wwp-1* | *ador-1* | *y37e3.8* | *rps-26* | *Y50D4C.3* |
| 19 | *rack-1* | *isp-1* | *acs-18* | *W02B3.7* | *rps-17* | *W02H5.2* |
| 20 | *rps-17* | *ztf-7* | *acr-3* | *rps-15* | *rpl-14* | *w02g9.5* |
| 21 | *rpl-36* | *gop-3* | *ah9.4* | *rps-5* | *rpl-24.1* | *pro-3* |
| 22 | *rpl-35* | *vha-9* | *w02g9.5* | *rack-1* | *rps-16* | *unc-11* |
| 23 | *eef-1A.1* | *atp-4* | *acr-11* | *Y116A8C.33* | *rpl-27* | *lin-38* |
| 24 | *rps-25* | *hsp-90* | *apc-17* | *zk1055.5* | *rpl-15* | *Y70C5B.1* |
| 25 | *rps-4* | *rpl-9* | *acr-24* | *che-13* | *asp-1* | *twk-45* |
| 26 | *f59d6.6* | *vha-4* | *arl-6* | *rpl-21* | *rpl-35* | *F11F1.1* |
| 27 | *rpl-34* | *gsr-1* | *f59d6.2* | *srh-174* | *rpl-31* | *mpst-2* |
| 28 | *Y45F10C.1* | *par-5* | *y39b6a.22* | *sra-11* | *rpl-5* | *srh-122* |
| 29 | *rps-19* | *rpn-10* | *adt-1* | *rpl-27* | *rps-15* | *tbc-13* |
| 30 | *rpl-31* | *ZK829.7* | *zk384.6* | *srbc-76* | *rpl-32* | *Y50D4A.4* |
| 31 | *rps-6* | *arx-3* | *aexr-1* | *rpl-9* | *rps-8* | *C27F2.1* |
| 32 | *rpl-30* | *rab-5* | *abt-1* | *rps-9* | *rps-28* | *Y95B8A.8* |
| 33 | *rps-0* | *sars-1* | *r09e10.4* | *rps-2* | *rpl-2* | *glb-25* |
| 34 | *Y7A5A.3* | *cey-1* | *aex-2* | *c08b6.5* | *y82e9br.3* | *sos-1* |
| 35 | *str-156* | *eif-1.A* | *ah10.2* | *rps-4* | *col-125* | *srh-174* |
| 36 | *rps-21* | *cap-2* | *arg-1* | *gcy-33* | *far-2* | *pqn-41* |
| 37 | *rpl-19* | *nap-1* | *acr-14* | *rla-1* | *rpl-43* | *ceh-14* |
| 38 | *rpl-15* | *prdx-2* | *t22b3.2* | *rps-3* | *rpl-13* | *tftc-1* |
| 39 | *rps-23* | *rpt-1* | *alh-2* | *rps-10* | *rps-14* | *y71h2am.3* |
| 40 | *rpl-5* | *f23h11.5* | *b0228.9* | *glb-9* | *rpl-34* | *C14B4.2* |
| 41 | *rps-5* | *ZK809.3* | *amt-2* | *rps-30* | *rps-12* | *F57C7.4* |
| 42 | *rps-8* | *sdhb-1* | *arrd-9* | *rpl-33* | *rpl-4* | *ZK721.4* |
| 43 | *c37a2.7* | *ret-1* | *acr-2* | *rps-29* | *rps-23* | *vab-19* |
| 44 | *rpl-7* | *f01f1.15* | *aqp-5* | *y7a5a.8* | *cyc-2.1* | *str-140* |
| 45 | *rpl-25.2* | *ubh-3* | *b0302.4* | *tax-4* | *ant-1.1* | *sdz-34* |
| 46 | *rpl-21* | *Y69A2AR.18* | *arrd-5* | *srh-8* | *rpl-7* | *srh-203* |
| 47 | *math-48* | *ran-3* | *arrd-3* | *f56d5.9* | *y79h2a.3* | *Y116A8B.4* |
| 48 | *y40c7b.1* | *ZK858.6* | *acr-9* | *rps-17* | *f11a1.2* | *w04g5.7* |
| 49 | *t05f1.7* | *rps-23* | *acdh-8* | *y68a4b.3* | *k11h12.9* | *Y59A8B.8* |
| 50 | *rpl-41* | *rps-22* | *y39b6a.24* | *f58g1.8* | *f41f3.3* | *ntl-3* |
