# Supplementary material for: Systematic Identification of Housekeeping Genes Possibly Used as References in Caenorhabditis elegans by Large-Scale Data Integration
Source: Cells. 2020 Mar 24;9(3):786. doi: 10.3390/cells9030786 (PMC7140892; doi:10.3390/cells9030786)
Supplement: Supplementary file 1 [file cells-09-00786-s001.zip › SM/Supplementary Material S6-Result of gene enrichment analysis using MetaScape.docx]

**Table S6**. Result of gene enrichment analysis using MetaScape.

| **GO** | **Category** | **Description** | **Count** | **%** | **Log10(P)** | **Log10(q)** |
| --- | --- | --- | --- | --- | --- | --- |
| R-CEL-72706 | Reactome GS | GTP hydrolysis and joining of the 60S ribosomal subunit | 48 | 30.97 | -72.13 | -68.24 |
| GO:0042254 | GO BP | ribosome biogenesis | 20 | 12.90 | -11.68 | -9.13 |
| GO:0002181 | GO BP | cytoplasmic translation | 12 | 7.74 | -10.97 | -8.46 |
| GO:0042273 | GO BP | ribosomal large subunit biogenesis | 8 | 5.16 | -6.16 | -3.75 |
| GO:0007568 | GO BP | aging | 15 | 9.68 | -5.46 | -3.08 |
| GO:0060079 | GO BP | excitatory postsynaptic potential | 5 | 3.23 | -4.65 | -2.33 |
| GO:0030490 | GO BP | maturation of SSU-rRNA | 5 | 3.23 | -3.98 | -1.71 |
| GO:0043254 | GO BP | regulation of protein complex assembly | 6 | 3.87 | -3.52 | -1.28 |
| R-CEL-622312 | Reactome GS | Inflammasomes | 4 | 2.58 | -3.16 | -0.96 |
| R-CEL-1227986 | Reactome GS | Signaling by ERBB2 | 3 | 1.94 | -2.64 | -0.54 |
| R-CEL-5689603 | Reactome GS | UCH proteinases | 4 | 2.58 | -2.55 | -0.46 |
| R-CEL-6791226 | Reactome GS | Major pathway of rRNA processing in the nucleolus and cytosol | 4 | 2.58 | -2.04 | -0.08 |

Reactome GS: Reactome Gene Set; GO BP: GO Biological Process
